# How well do coverage surveys and programmatically reported mass drug administration coverage match? Results from 214 mass drug administration campaigns in 15 countries, 2008–2017

**DOI:** 10.1136/bmjgh-2022-011193

**Published:** 2023-05-04

**Authors:** Kathryn L Zoerhoff, Pamela S Mbabazi, Katherine Gass, John Kraemer, Brian B Fuller, Lynsey Blair, Roland Bougma, Aboulaye Meite, Nebiyu Negussu, Bizuayehu Gashaw, Scott D Nash, Nana-Kwadwo Biritwum, Jean Frantz Lemoine, Helena Ullyartha Pangaribuan, Eksi Wijayanti, Karsor Kollie, Clara Fabienne Rasoamanamihaja, Lazarus Juziwelo, Square Mkwanda, Pradip Rimal, Issa Gnandou, Bocar Diop, Ameyo Monique Dorkenoo, Rachel Bronzan, Edridah Muheki Tukahebwa, Fatima Kabole, Violetta Yevstigneyeva, Donal Bisanzio, Lauren Courtney, Joseph Koroma, Egide Endayishimye, Richard Reithinger, Margaret C Baker, Fiona M Fleming

**Affiliations:** 1 International Development Group, RTI International, Washington, District of Columbia, USA; 2 The Task Force for Global Health, Decatur, Georgia, USA; 3 World Health Organization, Geneva, Switzerland; 4 School of Health, Georgetown University, Washington, District of Columbia, USA; 5 Helen Keller International, New York, New York, USA; 6 SCI Foundation, London, UK; 7 Ministère de la Santé et de l'Hygiène Publique, Ouagadougou, Burkina Faso; 8 Ministère de la Santé et de l'Hygiène Publique, Abidjan, Côte d'Ivoire; 9 Federal Ministry of Health, Addis Ababa, Addis Ababa, Ethiopia; 10 Bahir Dar University, Bahir Dar, Amhara, Ethiopia; 11 The Carter Center, Atlanta, Georgia, USA; 12 Ministry of Health, Accra, Ghana; 13 Ministère de la Santé Publique et de la Population, Port-au-Prince, Haiti; 14 Ministry of Health, Jakarta, Indonesia; 15 Ministry of Health, Monrovia, Liberia; 16 Ministère de la Santé Publique, Antananarivo, Madagascar; 17 Ministry of Health, Lilongwe, Malawi; 18 Ministry of Health and Population, Kathmandu, Nepal; 19 Ministère de la Santé Publique, de la Population, et des Affaires Sociales, Niamey, Niger; 20 Ministère de la Santé et de l'Action Sociale, Dakar, Senegal; 21 Ministère de la Santé, de l'Hygiène Publique, et de l'Accès Universel aux Soins, Lomé, Togo; 22 FHI 360, Washington, District of Columbia, USA; 23 Ministry of Health, Kampala, Uganda; 24 Zanzibar Ministry of Health, Stone Town, Tanzania, United Republic of; 25 USAID, Washington, District of Columbia, USA

**Keywords:** onchocerciasis, trachoma, filariasis, intervention study, public health

## Abstract

**Introduction:**

Delivering preventive chemotherapy through mass drug administration (MDA) is a central approach in controlling or eliminating several neglected tropical diseases (NTDs). Treatment coverage, a primary indicator of MDA performance, can be measured through routinely reported programmatic data or population-based coverage evaluation surveys. Reported coverage is often the easiest and least expensive way to estimate coverage; however, it is prone to inaccuracies due to errors in data compilation and imprecise denominators, and in some cases measures treatments offered as opposed to treatments swallowed.

**Objective:**

Analyses presented here aimed to understand (1) how often coverage calculated using routinely reported data and survey data would lead programme managers to make the same programmatic decisions; (2) the magnitude and direction of the difference between these two estimates, and (3) whether there is meaningful variation by region, age group or country.

**Methods:**

We analysed and compared reported and surveyed treatment coverage data from 214 MDAs implemented between 2008 and 2017 in 15 countries in Africa, Asia and the Caribbean. Routinely reported treatment coverage was compiled using data reported by national NTD programmes to donors, either directly or via NTD implementing partners, following the implementation of a district-level MDA campaign; coverage was calculated by dividing the number of individuals treated by a population value, which is typically based on national census projections and occasionally community registers. Surveyed treatment coverage came from post-MDA community-based coverage evaluation surveys, which were conducted as per standardised WHO recommended methodology.

**Results:**

Coverage estimates using routine reporting and surveys gave the same result in terms of whether the minimum coverage threshold was reached in 72% of the MDAs surveyed in the Africa region and in 52% in the Asia region. The reported coverage value was within ±10 percentage points of the surveyed coverage value in 58/124 of the surveyed MDAs in the Africa region and 19/77 in the Asia region. Concordance between routinely reported and surveyed coverage estimates was 64% for the total population and 72% for school-age children. The study data showed variation across countries in the number of surveys conducted as well as the frequency with which there was concordance between the two coverage estimates.

**Conclusions:**

Programme managers must grapple with making decisions based on imperfect information, balancing needs for accuracy with cost and available capacity. The study shows that for many of the MDAs surveyed, based on the concordance with respect to reaching the minimum coverage thresholds, the routinely reported data were accurate enough to make programmatic decisions. Where coverage surveys do show a need to improve accuracy of routinely reported results, NTD programme managers should use various tools and approaches to strengthen data quality in order to use data for decision-making to achieve NTD control and elimination goals.

**WHAT IS ALREADY KNOWN ON THIS TOPIC?**

- Ministries of health regularly monitor the programmatic performance of mass drug administration (MDA) and whether treatment coverage threshold targets set for various neglected tropical diseases (NTDs) have been achieved: this is typically done through the coverage that is reported as MDAs are being implemented, or post-MDA population-based coverage evaluation surveys—however, due to the different approaches how the data are collected, discrepancies in treatment coverages are common and may make programmatic decision-making difficult.

**WHAT THIS STUDY ADDS?**

- To understand the discrepancies of routinely reported treatment coverage and coverage estimated by coverage evaluation surveys, and how these discrepancies may affect programmatic decision-making, we analysed data from 214 NTD MDAs implemented between 2008 and 2017 in 15 countries in Africa, Asia and the Caribbean.
- Coverage estimates using routine reporting and surveys gave the same result in terms of whether a minimum disease-specific coverage threshold was reached in 72% of the MDAs surveyed in the Africa region and in 52% in the Asia region; the reported coverage value was within ±10 percentage points of the surveyed coverage value in 58/124 of the surveyed MDAs in the Africa region and 19/77 in the Asia region.
- Variation across countries in the number of surveys conducted as well as the frequency with which there was concordance between the two coverage estimates were observed.

**HOW THIS STUDY MIGHT AFFECT RESEARCH, PRACTICE OR POLICY?**

- For many of the MDAs surveyed, based on the concordance with respect to reaching the minimum coverage thresholds, the routinely reported data were accurate enough to make programmatic decisions.
- Where coverage surveys do show a need to improve accuracy of routinely reported results, NTD programme managers should use various tools and approaches to strengthen data quality in order to use data for decision-making to achieve NTD control and elimination goals.

## Introduction

Neglected tropical diseases (NTDs) cause substantial morbidity and mortality, and can result in great cognitive, social, emotional and economic harm.^1–6^ Therefore, accelerating progress towards their control and elimination could majorly contribute to reaching the Sustainable Development Goal to ‘ensure healthy lives and promote well-being for all.’^5 7^ For seven of the NTDs—lymphatic filariasis (LF), onchocerciasis (OV), schistosomiasis (SCH), three types of soil-transmitted helminths (STH) and blinding trachoma—delivering preventive chemotherapy (PC) through mass drug administration (MDA) is a central component to preventing morbidity and reducing transmission.^6 8 9^ Approximately one-fifth of the world’s population requires PC for at least one NTD^10^ in order to achieve either their control or elimination as public health problems in the next decade.^6^

MDA involves administering medication to at-risk populations, typically once or twice per year. Populations requiring MDA vary by disease and local epidemiology, ranging from pre-school and/or school-aged children to women of childbearing age to the full population. Treatment coverage thresholds set by WHO vary by disease: ≥65% of the total population for LF, ≥75% of school-age children for SCH and STH, ≥80% of the total population for trachoma, and≥65% of the total or ≥80% of the eligible population for OV.^11 12^ Regardless, achieving adequate treatment coverage is a prerequisite for meeting control and elimination goals in a timely, efficient and cost-effective manner, and can be part of the criteria to determine when it is appropriate to conduct surveys to show whether MDA can be stopped or its frequency reduced.^13 14^ Low treatment coverage wastes valuable resources if additional years of MDA are required, and potentially negates the progress towards achieving established NTD road map 2030 goals.^6 15 16^

Therefore, estimates of programme coverage need to be accurate *enough* to support programmatic decisions. These decisions include identifying low coverage districts for increased attention and targeted support, and determining when to conduct impact surveys to potentially stop or change MDA frequency. For any health intervention coverage data to be actionable, it needs to be valid and reliable, available in a timely fashion, and collected within real-world financial and logistical constraints—features which often trade off with one another.^17^

MDA treatment coverage, that is, the proportion of those who need treatment who received and actually swallowed a dose, can be measured using two different sources of data: data from routine programmatic data or from specially implemented, population-representative coverage evaluation surveys.^12^ Coverage calculated using routinely reported data uses administrative data on the number of persons treated, as recorded by drug distributors during MDAs for the numerator. For the denominator, existing population estimates, normally from national census or programme registers, are used. Use of administrative data enables coverage estimates to be made at granular—district and subdistrict—levels, for each MDA, every year. However, it is potentially susceptible to denominator errors in estimated population size, which can be exacerbated when only subpopulations are targeted for treatment.^18–24^ Furthermore, if drug distributors do not directly observe patients taking MDA doses, the reported coverage may overestimate the fraction of the population that actually receive and swallow preventive chemotherapy.^25–28^ There is often a lack of trust in the quality of reported coverage due to limited arithmetic skills among some drug distributors, errors in counting total numbers treated in collated paper-based reports and registers, possible incentives for intentionally inflating the data and difficulty capturing treatments occurring outside the national programme (eg, through the private sector or non-governmental organisations).^18–22 24 29^ Anecdotally, national NTD programme managers sometimes report challenges due to insufficient/inappropriate data collection tools (eg, there may be treatment registers but no printed summary forms for summarising data).

Alternatively, treatment coverage can be estimated by conducting community-based coverage evaluation surveys. Coverage estimates based on surveyed data are often believed to produce more valid results, and WHO currently recommends that surveys be used to periodically assess the quality of reported coverage.^12 14^ However, coverage surveys are logistically more complicated and require additional resources and more complex analysis than reported coverage, with estimates usually being available to programme managers less quickly. In addition, coverage evaluation surveys are often designed to be representative at the district level,^12^ which precludes estimates in small but programmatically important administrative units, such as subdistricts; surveyed coverage may also be susceptible to selection and information bias^30^ such as recall bias (although one study did find that recall was fairly accurate up to 1 year post-MDA).^31^ Lastly, there is concern that surveys might be missing the same populations as MDAs, including because people are not at home due to work obligations that can take them away for days or weeks at a time, or because populations to be treated are nomadic and vulnerable populations that may not have fixed or permanent abodes.

The objective of the analyses presented here was to evaluate concordance between reported and surveyed coverage of MDAs conducted in 15 countries between 2008 and 2017. We incorporated coverage estimates as calculated by countries because these are the estimates from which programme managers would make programmatic decisions. Specifically, we aimed to understand (1) how often coverage calculated using routinely reported data and survey data would lead programme managers to make the same programmatic decisions, (2) the magnitude and direction of the difference between these two estimates, and (3) whether there is meaningful variation by region, age group, or country.

## Methods

### Data sources and population

The data we analyse in this paper are data collected and used by NTD programme managers for programmatic decision making across 15 countries between 2008 and 2017. MDAs and coverage surveys were implemented by country Ministries of Health (MOHs), with the support of various NTD technical implementing partners (ie, RTI International, SCI Foundation, the Taskforce for Global Health, FHI360, Health & Development International and The Carter Center) and funded by several bilateral donors (ie, the U.S. Agency for International Development, the U.K. Department for International Development, END Fund, Children’s Investment Fund Foundation and private donors).

We evaluated the concordance between data collected from two sources: routinely reported epidemiological coverage data and coverage survey data. While using these sources limits the quality of the data, it provides access to data on a much larger scale than routinely available to academic research projects, and allows exploration of patterns and trends in existing data used to influence how programmatic decisions are made.

#### Reported coverage

Routinely collected reported coverage was compiled using data reported by national NTD programmes to donors, either directly or via NTD implementing partners. NTD programmes collect PC treatment data from drug distributors, using either a community register or a tally sheet, and then these data are aggregated on summary forms and submitted to the next administrative level within the respective MOH (or Ministry of Education) reporting system. Coverage is calculated by dividing the number of individuals treated by a population value (see table 1 for description of types of coverage, definition and targets). Typically, the source of these population values was national census projections or, in some cases, the community registers. More detailed descriptions of how data are collected, checked for quality, analysed and reported in order to produce estimates of reported coverage have previously been published.^32–34^

**Table 1:** Key types of MDA treatment coverage, their definition and specific preventable chemotherapy neglected tropical disease coverage threshold targets

Type of coverage	Definition	Target
Geographical coverage	No of endemic implementation units where PC is implemented/no of endemic implementation units where PC is required	100% for all diseases
National coverage	No of individuals ingesting PC medicines in an endemic country/population living in all implementation units where PC is required	≥65% for LF
		≥65% for control of OV, ≥80% for elimination of OV
		≥75% of school-age children (SAC) for SCH
		≥75% for STH
		≥80% for trachoma
Epidemiological coverage	No of individuals ingesting PC medicines at implementation unit level/population living in an implementation unit where PC is implemented	≥65% of total population for LF
		≥65% of total population for control of OV, ≥80% of total population for elimination of OV
		≥75% of SAC for SCH
		≥75% of pre-SAC and SAC for STH
		≥80% of total population for trachoma
Programme coverage	No of individuals ingesting the PC medicines/eligible population targeted for treatment in implementation unit where PC is implemented	≥75% of eligible population targeted for SCH, STH or ≥80% of eligible population targeted for LF, OV, trachoma
Surveyed coverage	No of surveyed individuals ingesting PC medicines at implementation unit level/population surveyed	see above for disease-specific targets

LF, lymphatic filariasis; MDA, mass drug administration; OV, onchocerciasis; PC, preventable chemotherapy; SCH, schistosomiasis; STH, soil-transmitted helminths.

#### Surveyed Coverage

The coverage surveys were conducted in support of national, government-led NTD programmes following MDAs that had been implemented (table 2). Our unit of analysis is district-level MDA surveyed coverage values, with each disease counting as a separate survey instance. In 24 districts assessed, school-age children and adults were surveyed separately. A total of 319 disease-specific coverage rates were assessed in this manner. To be included in the analyses, the following information was needed for each MDA surveyed: country and district name, year of survey, age group(s) surveyed, disease surveyed, reported epidemiological coverage, surveyed coverage point estimate and 95% CIs. In addition to excluding implementation units (IUs) which had incomplete information, we also excluded MDAs for which coverage surveys were insufficiently powered, defined as CIs greater than ±10 percentage points based on what is thought to be required precision to inform operational and management decisions.

**Table 2:** MDAs and districts surveyed, by region, country, disease, year and age groups

Region	Country	Disease(s) surveyed	Year	Age group(s) surveyed	Number of districts surveyed*	Number of MDAs surveyed†
Africa	Burkina Faso	LF	2015	All	2	2
Africa	Cote d’Ivoire	SCH	2014	SAC	4	4
		SCH	2016	SAC	1	1
Africa	Ethiopia	SCH, STH	2015	SAC	8	16
		SCH, STH	2016	SAC	6	12
		STH	2016	SAC	3	3
		SCH, STH	2017	SAC	7	14
		TR	2017	All	4	4
Africa	Ghana	LF	2009	All	7	7
Africa	Liberia	SCH	2017	SAC	1	1
Africa	Madagascar	SCH, STH	2016	SAC	3	6
		SCH	2016	SAC	1	1
		SCH, STH	2017	SAC	1	2
		SCH	2017	SAC	1	1
Africa	Malawi	SCH	2012	SAC	1	1
		SCH	2014	SAC	6	6
		LF	2014	All	2	2
		SCH, STH	2016	SAC and adults (for both SCH and STH)	1	4
		SCH, STH	2016	SAC and adults for SCH, SAC only for STH	1	3
		SCH, STH	2016	SAC	1	2
		STH	2016	SAC	1	1
		SCH	2016	SAC	2	2
Africa	Niger	TR	2015	All	4	4
		SCH	2017	SAC	2	2
Africa	Senegal	LF	2015	All	8	8
Africa	Togo	OV, SCH, STH	2012	SAC for SCH and STH, all for OV	2	6
		OV, STH	2012	SAC for STH, all for OV	1	2
Africa	Uganda	LF	2008	All	3	3
Africa	Zanzibar	SCH	2015	SAC and adults	2	4
Asia	Indonesia	LF	2015	All	7	7
		LF	2016	All	12	12
		LF	2017	All	6	6
Asia	Nepal	LF	2013	All	10	10
		LF	2014	All	21	21
		LF	2015	All	21	21
Americas	Haiti	LF	2012	All	6	6
		LF	2013	All	7	7
TOTAL					176	214

*Refers to Administrative Level 2 units. If a district was surveyed for multiple diseases in a given year, it is counted as one district. If a district was surveyed over multiple years, it was counted each year; for example, if the same district was surveyed in 2016 and 2017, it was counted once in 2016 and once in 2017.

†Number of district-level MDAs with survey coverage estimates. Counts each disease, district and age group surveyed as a separate instance; for example, if a district was treated for SCH and STH, and the survey assessed SCH and STH coverage, it is counted twice since the results could have differed between SCH and STH.

LF, lymphatic filariasis; MDA, mass drug administration; OV, onchocerciasis; SAC, school-age children; SCH, schistosomiasis; STH, soil-transmitted helminths; TR, trachoma.

In some cases, the IUs surveyed were randomly selected, while in other cases, they were purposively selected based on high or low reported coverage, number of years of programme implementation, a high number of adverse events and/or concerns about the accuracy of reported coverage. The coverage surveys were typically carried out between 1 and 6 months after the MDA. The number of clusters randomly selected in each IU varied by survey. Households to be surveyed were selected using country-determined strategies, such as the spin-the-bottle and then random walk method used by the Expanded Programme on Immunization,^33^ a line listing or enumeration of all village households and then selecting households using systematic sampling without replacement,^32^ or a modified segmentation approach.^35^ Countries then followed their own processes to estimate and submit surveyed coverage values and CIs; we used these values, as reported by national NTD programmes, because they represent the information available to NTD programme managers.

### Data analysis

To assess the differences between routine epidemiological programme and survey coverage estimates, we made two comparisons. First, we assessed how often the coverage estimates were on different sides of a critical MDA treatment coverage threshold (table 1): 65% for LF and OV, 75% of school-age children for SCH and STH, and 80% for trachoma, in order to examine the extent to which reported coverage would lead to the same decision-making as surveyed coverage. To do this, we categorised the reported and surveyed coverage values as concordant or discordant with respect to a respective disease’s target coverage threshold. Thus, if both the reported and surveyed coverage values were above or both were below the target threshold (ie, they would lead to the same programmatic decision-making), such outcome was considered concordant. Conversely, if one of the values was above and the other below coverage thresholds, such outcome was considered discordant. CIs were not taken into consideration for this exercise because we assumed that surveys were powered to a degree of precision appropriate for decision-making. Second, we assessed how often the two coverage estimates were within 10 percentage points of each other. Ten percentage points was selected because those differences are small enough that most managers would consider the discrepancy immaterial to influence programmatic decisions. We identified the proportion of MDAs where reported coverage was at least 10 percentage points greater than the surveyed coverage point estimate, and the proportion with reported coverage at least 10 percentage points less than surveyed coverage.

In order to account for variation in concordance by region (Africa or Asia), country and age group surveyed (total population, school-age children only), we conducted analyses at these disaggregated levels. We also assessed the concordance between reported and surveyed point estimates of MDA coverage using the Lin’s concordance test. The Lin’s ρ ranges from −1 to 1, with perfect agreement at ρ=1.^36^ The area under the curve (AUC) calculated using the receiver operating characteristic curve was used to compare agreement between the two coverage estimates regarding being above a disease’s target coverage threshold (dichotomous variable: 1=above threshold, 0=below threshold). An AUC <0.7 is generally considered as low, 0.7 to 0.8 as acceptable, 0.8 to 0.9 as excellent and >0.9 as perfect.^37^ All analyses were performed using the R language, V.3.6.2.

Data in this study were not weighted by country, as the unit of analysis is the district-level MDA coverage values, which represent the decision points that programme managers would use to base concordance or discordance.

### Patient and public involvement statement

The data analysed originated from routine NTD programmatic efforts, and they were analysed in aggregate. As a result, it was not appropriate or possible to involve patients or the public in the design, or conduct, or reporting, or dissemination plans of our research. The public will benefit from the findings of our study as—depending on the country data—NTD efforts will be maintained and or expanded to ensure programmatic coverage thresholds will be reached. The authorship team on this paper is composed primarily of MOH NTD programmes and implementing partner monitoring and evaluation staff, who worked together closely in the design and implementation of MDAs and surveys, the surveys’ data analysis and in the interpretation of results—this is the perspective and experience that informed the genesis, analyses and writing of this paper.

### Ethical considerations

MDAs and the reported epidemiological coverage are routine health interventions and do not undergo ethical review. Depending on the country, MOHs determined in alignment with country policies whether the protocol for coverage surveys required institutional review board or other ethical approval; verbal consent was obtained from each survey respondent or an adult on behalf of young children prior to administering a coverage survey questionnaire.

This study only used existing (secondary) data that were collected for public health planning and programming purposes. The data that were shared by country programmes were only available and analysed at the aggregate level and thus ethical approval for the analyses was not necessary a priori.

## Results

**Figure 1:**
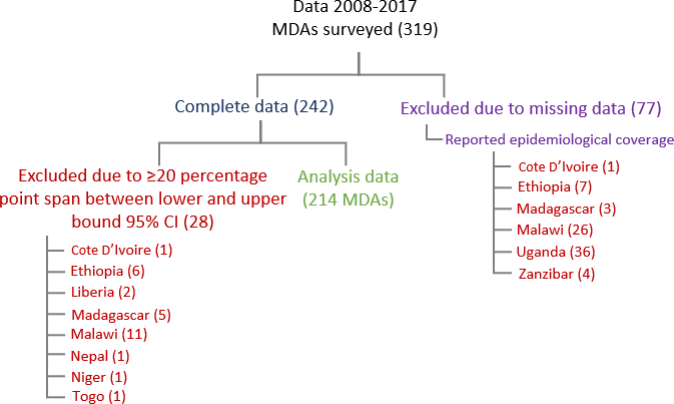
PRISMA flow diagram of MDAs and surveys included in the analyses. MDA, mass drug administration.

Coverage data on 319 MDAs conducted between 2008 and 2017 were available (figure 1). For 77 of these MDAs, there was incomplete coverage survey data, with 62 (80.5%) coming from two countries (ie, Malawi and Uganda). Similarly, data on 28 MDAs were excluded as the CIs were greater than ±10 percentage points; 22 (78.6%) of these came from three countries (ie, Ethiopia, Madagascar and Malawi). Consequently, coverage data on 214 disease-specific MDAs were included in our analyses, with data assessing programmatic NTD efforts in 176 districts across 15 countries (table 2).

Of the 214 MDAs with epidemiological and survey coverage data included in our analyses, 124 were in Africa and 77 were in Asia. The number of coverage surveys implemented per country ranged from 1 to 52. When surveys were implemented across multiple years, most countries selected new districts to survey; however, in Indonesia, Malawi and Nepal, there were 3, 3 and 9 districts, respectively, that were surveyed multiple times. Frequently, NTD programmes leveraged integrated MDA platforms and surveyed multiple diseases in any given district; for example, 28 districts were surveyed for both SCH and STH. Our analysis included 112 MDAs assessed for LF, 3 for OV, 55 for SCH, 36 for STH and 8 for trachoma.

**Figure 2:**
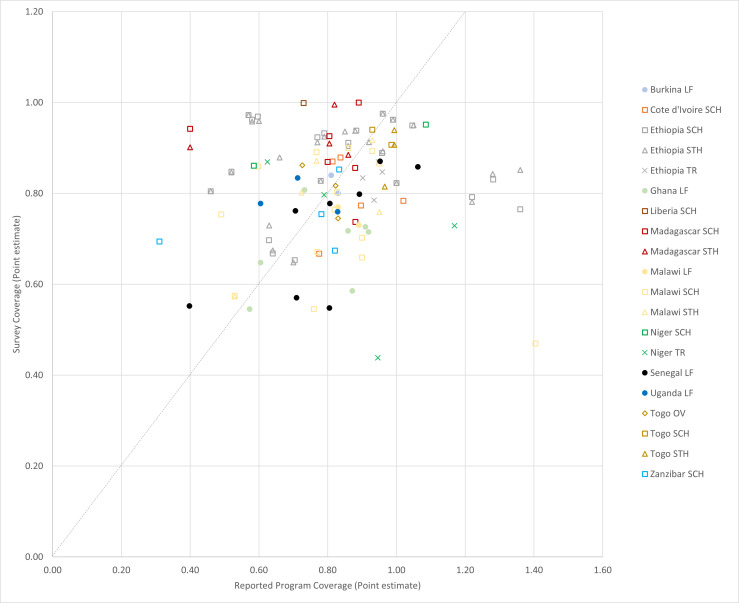
Reported vs surveyed coverage in 124 MDAs surveyed in Africa, 2008–2017. LF, lymphatic filariasis; MDA, mass drug administration; OV, onchocerciasis; SAC, school-age children; SCH, schistosomiasis; STH, soil-transmitted helminths; TR, trachoma.

In the districts surveyed in the Africa region, there was concordance (agreement) in coverage estimates from routine and surveyed sources with respect to the target coverage threshold 72% of the time (table 3), with the AUC equal to 0.638 (online supplemental figure 1). The reported coverage value was within ±10 percentage points of the surveyed coverage value for 47% (58/124) of the included MDAs (table 3, figure 2). Of the surveyed districts in the Africa region, 27% (33/124) showed underreporting, defined as surveyed coverage results at least 10 percentage points higher than reported. Similarly, the same number of districts (33) showed overreporting, that is, surveyed coverage was at least 10 percentage points lower than reported. The correlation between reported and surveyed coverage in Africa was very low, with a Lin’s ρ=0.08 (95% CI −0.08, 0.23).

10.1136/bmjgh-2022-011193.supp1 Supplementary data

**Table 3:** 

			Concordant decision-making with respect to target coverage threshold		Agreement (%)	Discordant decision-making with respect to target coverage threshold		Underreporting	Overreporting
Group†	Disaggregation	Number of MDAs surveyed	Number (%) of MDAs with both reported and surveyed coverage below the threshold	Number (%) of MDAs with both reported and surveyed coverage above the threshold		Number (%) of MDAs with reported coverage above threshold and surveyed below	Number (%) of MDAs with surveyed coverage above threshold and reported below	Number (%) of MDAs with surveyed coverage ≥10 percentage points greater than reported	Number (%) of MDAs with reported coverage ≥10 percentage points greater than surveyed
Total	Total	214	21 (10)	122 (57)	67	51 (24)	20 (9)	33 (15)	96 (45)
Region	Africa	124	13 (10)	77 (62)	72	14 (11)	20 (16)	33 (27)	33 (27)
	Asia	77	8 (10)	32 (42)	52	37 (48)	0 (0)	0 (0)	58 (75)
Age group	Total population	123	12 (10)	66 (54)	64	43 (35)	2 (2)	5 (4)	75 (61)
	SAC	86	6 (7)	56 (65)	72	6 (7)	18 (21)	27 (31)	19 (22)
Country	Burkina Faso	2	0 (0)	2 (100)	100	0 (0)	0 (0)	0 (0)	0 (0)
	Cote d'Ivoire	5	0 (0)	4 (80)	80	1 (20)	0 (0)	0 (0)	3 (60)
	Ethiopia	49	6 (12)	31 (63)	75	1 (2)	11 (22)	15 (31)	11 (22)
	Ghana	7	2 (29)	4 (57)	86	1 (14)	0 (0)	0 (0)	4 (57)
	Haiti	13	0 (0)	13 (100)	100	0 (0)	0 (0)	0 (0)	5 (38)
	Indonesia	25	1 (4)	9 (36)	40	15 (60)	0 (0)	0 (0)	22 (88)
	Liberia	1	0 (0)	0 (0)	0	0 (0)	1 (100)	1 (100)	0 (0)
	Madagascar	10	0 (0)	7 (70)	70	1 (10)	2 (20)	6 (60)	1 (10)
	Malawi	21	2 (10)	11 (52)	62	5 (24)	3 (14)	4 (19)	6 (29)
	Nepal	52	7 (13)	23 (44)	57	22 (42)	0 (0)	0 (0)	36 (69)
	Niger	6	1 (17)	1 (17)	34	2 (33)	2 (33)	2 (33)	3 (50)
	Senegal	8	1 (13)	5 (63)	76	2 (25)	0 (0)	1 (13)	3 (38)
	Togo	8	0 (0)	8 (100)	100	0 (0)	0 (0)	1 (13)	1 (13)
	Uganda	3	0 (0)	2 (67)	67	0 (0)	1 (33)	2 (67)	0 (0)
	Zanzibar	4	1 (25)	2 (50)	75	1 (25)	0 (0)	1 (25)	1 (25)

*Sums of percentages may not equal 100% due to rounding.

†Age group and region breakdowns do not include adults surveyed or Latin American countries surveyed due to small samples when disaggregating.

**Figure 3:**
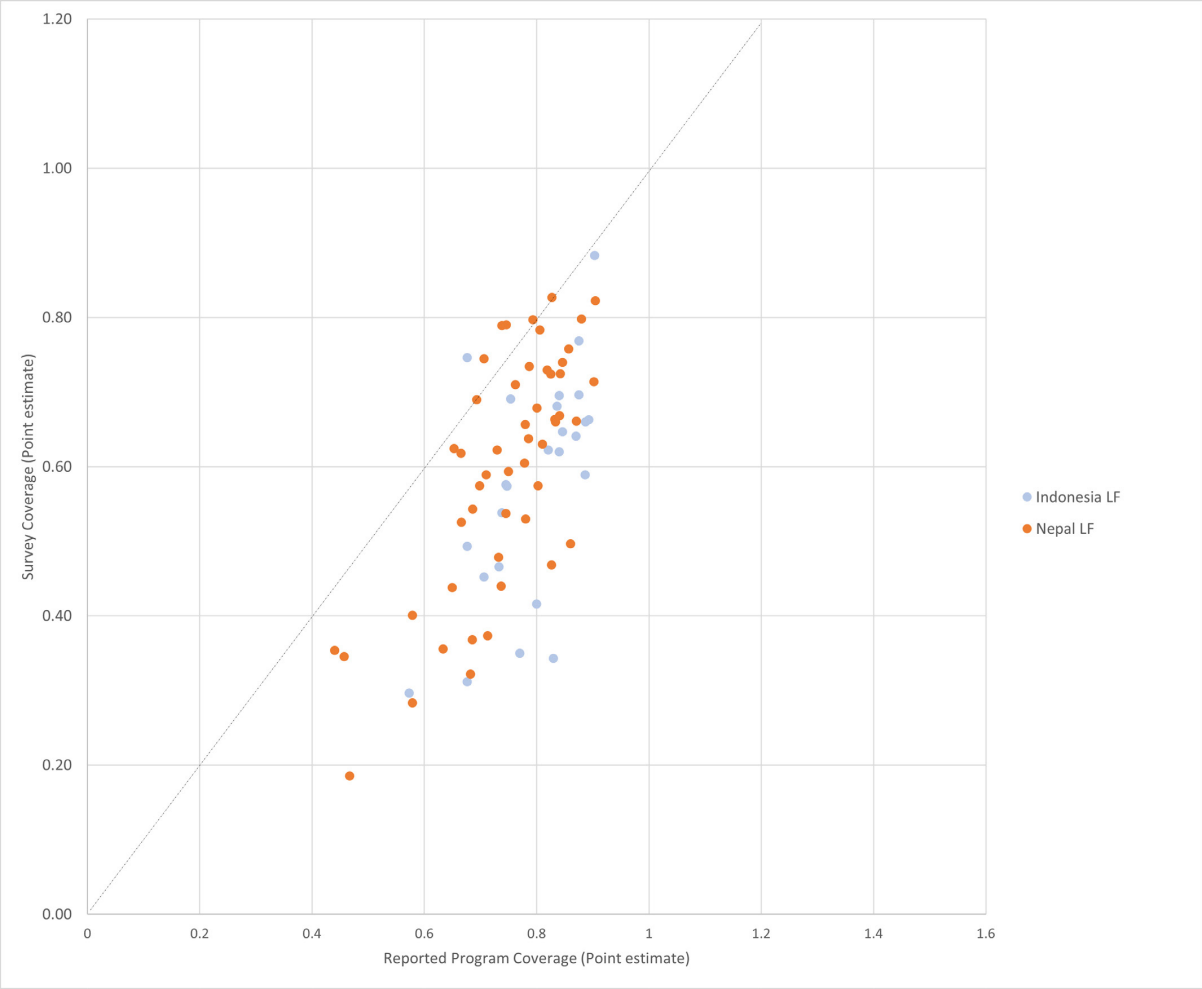
Reported vs surveyed coverage in 77 MDAs surveyed in Asia, 2008–2017. LF, lymphatic filariasis.

In the districts surveyed in the Asia region, there was concordance between coverage estimates 52% (40/77) of the time (table 3), with the AUC equal to 0.589 (online supplemental figure 1). The reported coverage value was within ±10 percentage points of the surveyed coverage value in 25% (19/77) of the MDAs surveyed (see figure 3). Of districts surveyed, 75% (58/77) showed that the routinely reported coverage was more than 10 percentage points higher than surveyed coverage (ie, overreporting), with none showing underreporting. The correlation between reported and surveyed coverage in Asia was low, with a Lin’s ρ=0.33 (95% CI 0.22, 0.43).

### By country

**Figure 4:**
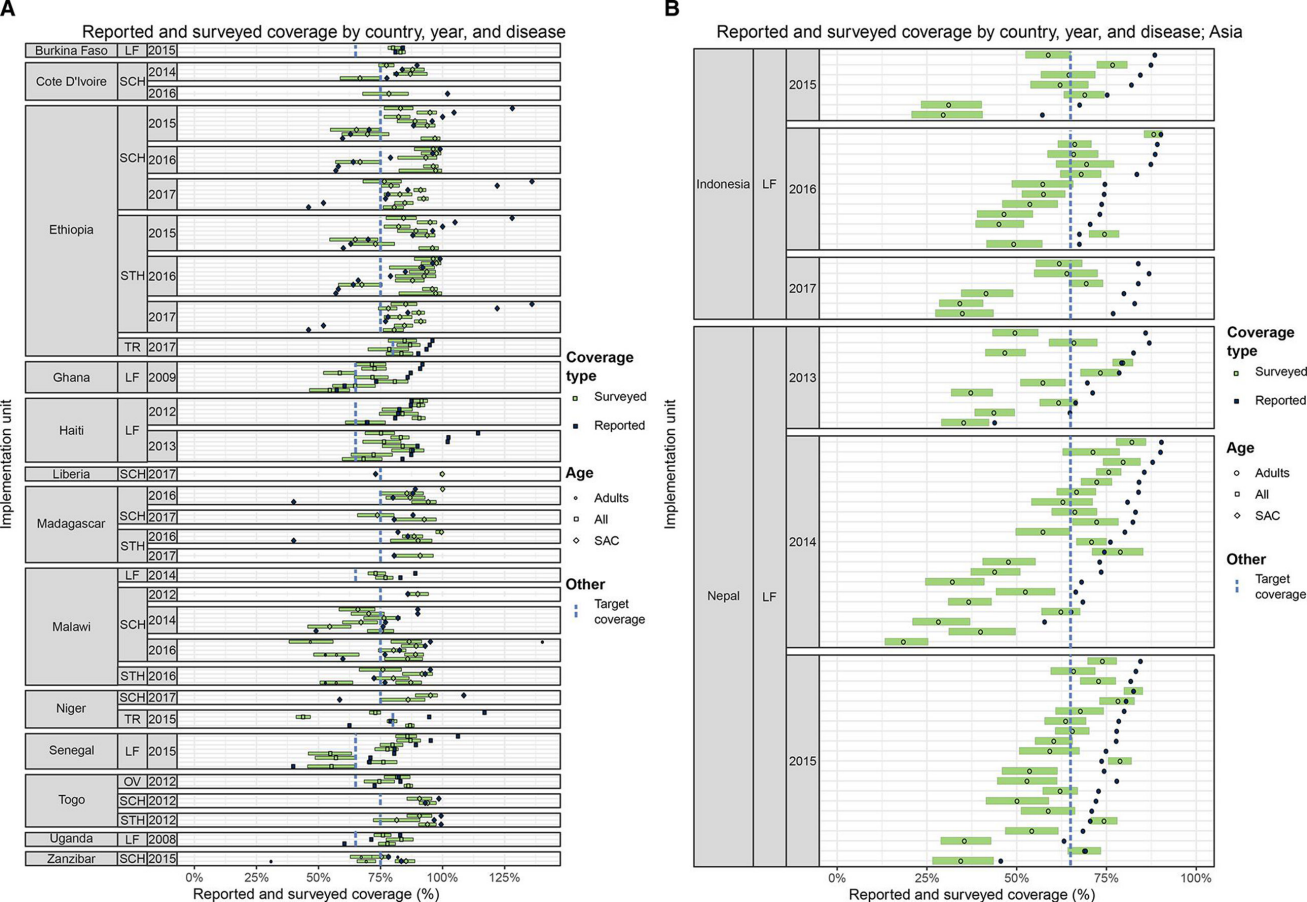
figure 4A Reported and surveyed coverage by country, year, and disease (Africa and Haiti). Green bars represent 95% CIs; LF, lymphatic filariasis; OV, onchocerciasis; SAC, school-age children; SCH, schistosomiasis; STH, soil-transmitted helminths; TR, trachoma. figure 4b Reported and surveyed coverage by country, year and disease (Asia). Green bars represent 95% CIs; LF, lymphatic filariasis; SAC, school-age children.

Results are shown by country in figure 4A and B. The median absolute difference between surveyed coverage and reported coverage in the implementation units assessed ranged from 3 percentage points in one country to 27 percentage points in another. Two main differences are observed among countries. First, there was a large difference in the number of surveys conducted by country, ranging from 1 to 52. Second, our analyses showed variation in the frequency with which there was concordance between the two coverage estimates, with some countries showing similar results between reported and surveyed coverage across most of the districts surveyed, others a heterogenous spread across districts surveyed, and yet others with mostly large discrepancies between the two coverage estimates. That said, it is important to use caution when interpreting the country-specific presented, as the districts surveyed may have been selected purposively and therefore are not representative of the entire country.

### By age group

Concordance between surveyed and routinely reported coverage estimates was 64% for the total population and 72% for school-age children (table 3). The reported coverage value was within ±10 percentage points of the surveyed coverage point estimate in 35% of the time for the total population, compared with 47% for school-age children. Only 4% of the districts that surveyed the total population showed underreporting, compared with 31% of the districts that surveyed school-age children. In contrast, in 61% of districts assessing the total population, the reported coverage was at least 10 percentage points higher than the surveyed coverage, while 22% of the MDAs assessing school-age children showed overreporting of more than 10 percentage points compared with the surveyed coverage point estimate.

## Discussion

The objective of the analyses presented here was to evaluate concordance between reported and surveyed coverage using results from MDAs conducted in 15 countries between 2008 and 2017, representing the largest published multi-country analysis of NTD coverage data to date. The analyses examined the level of concordance between district-level MDA coverage estimates calculated using available data from two sources: routinely reported data and coverage survey data.

In the countries included from the Africa region, there was agreement between the two coverage estimates in 72% of cases—in other words, in 72% of cases both routinely reported and surveyed coverage fell on the same side of the target MDA threshold and would have led to the same programmatic decision-making (ie, continuing or discontinuing MDA). This corroborates a prior study that had analysed reported and surveyed coverage estimates in Cameroon, Malawi, Mali, Nigeria and Togo, and that had observed a high degree of concordance between the two estimates.^33^ However, unlike our analyses, which observed no bias toward underreporting or overreporting in the Africa region, this prior study showed that where there were discrepancies between reported and surveyed coverage estimates, these tended towards overreporting.

Concordance between reported and surveyed coverage estimates was lower in Asia at 52%, with routinely reported results often overestimating the true coverage of persons swallowing treatment. This is also in line with other surveys conducted in the Asia region, which often indicate challenges with compliance and the frequent overreporting that occurs when directly observed therapy is not implemented.^33 38^ Indeed, in a systematic review of 36 studies published from India, Babu and Babu^38^ found a ‘coverage–compliance gap’, reporting that on average, people ingesting the drugs was 22% less than people receiving it. They argued that the most likely reason was that drug distributors were trying to cover a large number of houses in a short time and often left the tablets behind to be consumed later.

Between-country differences were also observed. Thus, some countries had conducted multiple surveys that mostly triangulated with reported coverage rates, and they could be sure that their routinely reported data was fit for purpose; others had done several surveys and large discrepancies were found, leading them to rely more heavily on the use of coverage surveys while seeking to improve routine data quality. Lastly, there were countries that had done only a small number of surveys and/or where results varied a lot within a country. This speaks to the importance of MDA programmes including coverage surveys as part of their routine programme monitoring and evaluation activities, particularly if the prevalence of infection and disease is not decreasing as expected following multiple rounds of MDA.

Less than half of surveyed estimates were within 10 percentage points of routinely reported coverage estimates among districts surveyed in Africa and only one quarter among districts surveyed in Asia. Similar findings have been reported from the immunisation field. For example, a comparison of reported vaccination coverage and coverage surveyed through the Demographic and Health Surveys (DHS) in 45 countries across multiple regions showed that three quarters of the estimates had more than a 10-percentage-point difference.^29^ Another analysis of DHS data found that there were discrepancies of at least 25 percentage points between reported and surveyed values in 10 out of the 44 countries analysed.^22^ Reasons for such discrepancies between reported and surveyed coverage estimates include data transcription errors, ability of respondents to recall drug consumption^31^ and that coverage surveys—similar to academic research studies—tend to be better resourced than routine, large-scale programmatic monitoring and evaluation efforts.

Ultimately, the purpose of MDA treatment coverage estimates is to enable NTD programme managers to determine approximate levels of coverage in order to make programmatic decisions, including if needed corrections. Despite appearing straightforward, this often requires nuanced and contextual interpretation of the data. For example, the results from the 13 districts surveyed in Haiti underscore the value of looking beyond the traditional interpretation of validating reported coverage, with the difference between reported and surveyed coverage in those districts ranging from 1 to 39 percentage points. Although only 8 of the 13 districts surveyed showed reported coverage was within 10 percentage points of the surveyed coverage point estimate, all of the 13 districts showed concordance with respect to the decision-making threshold of achieving at least 65% epidemiological coverage for LF. Transmission assessment surveys conducted since the coverage surveys showed that all 13 of the districts included in this study have achieved the criteria for stopping MDA (data not shown).

Programme managers must grapple with making decisions based on imperfect information, balancing needs for accuracy with cost and available capacity. Where coverage surveys do show the need to improve accuracy of routinely reported results, NTD programme managers have a few tools and approaches available that can be used for this purpose. For example, routinely reported data can be disaggregated to subdistrict levels to determine if there are missing data. WHO guidance is to use national census projections except in cases where the national census may not be updated, is considered inaccurate or may exclude certain populations, such as nomadic populations.^13^ For example, in Sierra Leone, a study found populations in two districts to be higher than projected from census data following post-conflict population growth due to reopening of mines resulting in post-war employment opportunities.^39^ If there are concerns about denominator accuracy, the population data can be triangulated using other data sources, such as health facility catchment area populations, community drug distributor pre-MDA registration, and remote sensing data using nightlights and land use.^21 39^ Furthermore, the quality of reported data can be reviewed during supportive supervision visits and data quality assessments to determine which aspects of the reporting system are not functioning well, and to identify actions to strengthen the system such as improved training on data collection, increased supervision or revising data collection tools.^12 40^

One opportunity for further research is to supplement the data included in this study with data from additional coverage evaluation surveys and look in more detail and differences based on MDA distribution mechanism (eg, school-based vs house-to-house drug administration) and of the drug used/disease targeted. There is also a need to further explore whether coverage surveys are missing the same populations missed by MDAs (eg, mobile and migrant populations).^41^ In addition, research can examine whether data quality improves as NTD data are becoming mainstreamed into national health information systems and/or moving into online, electronic data management platforms.

### Limitations

A number of potential caveats of our analyses should be highlighted, most of which are due to the fact that we used routinely collected programmatic MOH data in our analyses and not data from carefully controlled academic research studies. First, both routinely reported coverage and surveyed coverage were calculated by countries using their protocols, and there is variation from country to country. As a result, the data are invariably noisy. For example, 16 (7%) of MDAs included in our analyses have reported coverage levels greater than 100%—most likely due to inaccurate denominator estimates projected from national census data (see above). Nonetheless, the study analyses exactly the data that NTD programme managers have available to make programmatic decisions, which would not be the case if additional controls were imposed. Second, we did not analyse disaggregated data. Analysis of disaggregated data is important programmatically as it provides crucial insight into what groups are not being effectively reached (eg, geographical areas, specific age–sex groups, and mobile and migrant populations). Coverage surveys also usually collect important information on why people were not treated. There is a need for future studies that look at these data across national programmes, identifying trends and issues that lend themselves to a collaborative approach for developing solutions. Third, the MDAs and surveys for which coverage data were included in our analyses received substantial support, ranging from technical assistance by technical implementing partners to substantial operational and funding support—which may limit potential generalisability to countries with a similar country MOH and stakeholder landscape. Ultimately, for countries being able to reach MDA treatment coverage thresholds and have strong concordance between reported and survey coverage requires multiple factors, including a strong and well-resourced MOH and NTD programme at all administrative levels, local and international technical implementing partners that can support implementation of MDAs and surveys, communities that are accepting of and adhering to the treatment, and robust monitoring and evaluation systems.

## Conclusion

A common global health programming question is whether we can have any faith in the validity of routinely reported health intervention coverage results—NTD programming is no exception. Based on the data presented here, we conclude that, yes, indeed, in many cases, we can have faith. The advantage of routinely reported coverage estimates is that it can be available soon after MDAs, for each MDA, at district and subdistrict levels. However, our analyses also show that there are countries where surveyed coverage was often vastly divergent from reported results in the districts surveyed. In those instances, NTD programme managers are flying blind until they can improve the data quality of their programmatic reporting. Each national NTD programme should seek to triangulate their routinely calculated coverage estimates with other data sources, including separately conducted coverage surveys. Ultimately, by using routinely reported data as well as conducting coverage surveys, national NTD programmes will be better equipped to strengthen the quality of their programmes and make better and effective decisions to reach NTD country control and elimination goals.

## Data Availability

Data are available upon reasonable request.
