# Supplementary figures and images for: How well do coverage surveys and programmatically reported mass drug administration coverage match? Results from 214 mass drug administration campaigns in 15 countries, 2008–2017
Source: BMJ Glob Health. 2023 May 4;8(5):e011193. doi: 10.1136/bmjgh-2022-011193 (PMC10163531; doi:10.1136/bmjgh-2022-011193)

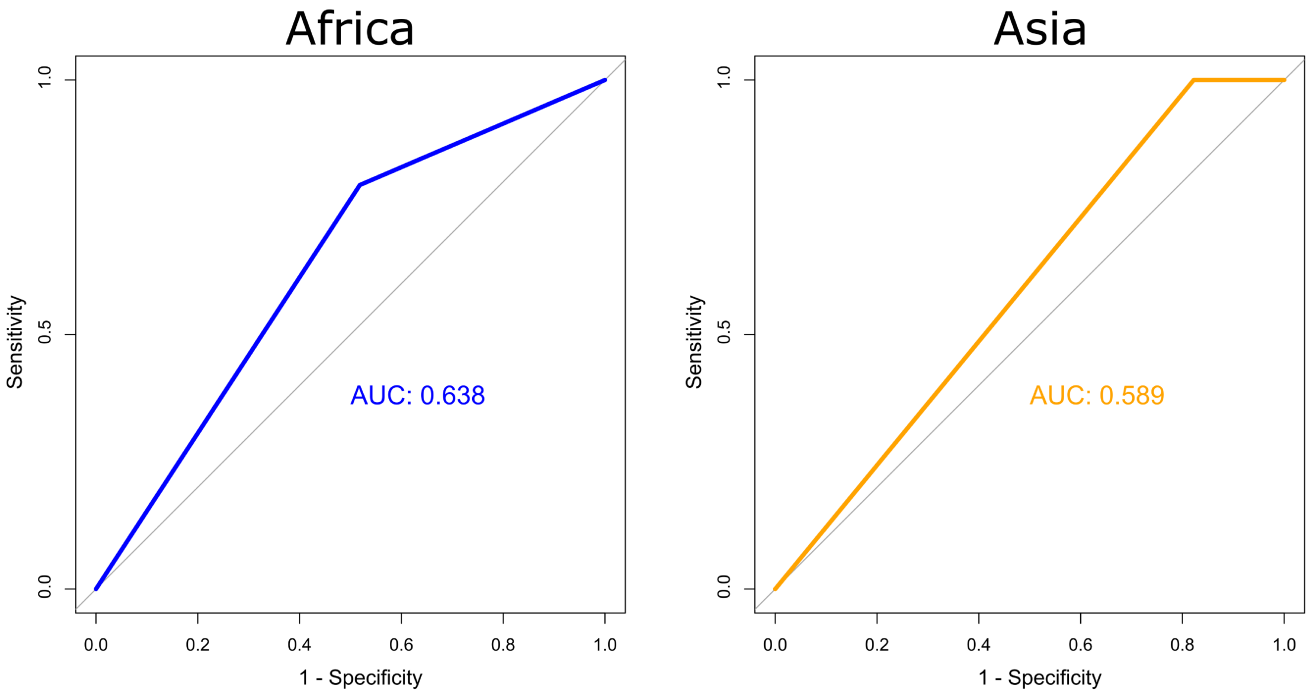

Supplement: Supplementary data [file bmjgh-2022-011193supp001.pdf]
